# A preoperative prediction model based on Lymphocyte-C-reactive protein ratio predicts postoperative anastomotic leakage in patients with colorectal carcinoma: a retrospective study

**DOI:** 10.1186/s12893-022-01734-5

**Published:** 2022-07-23

**Authors:** Bin Zhong, Zhen-Yu Lin, Dan-Dan Ma, Zuo-Hong Shang, Yan-Bin Shen, Tao Zhang, Jian-Xin Zhang, Wei-Dong Jin

**Affiliations:** 1 The First School of Clinical Medicine, Southern Medical University, Guangzhou, 510515 China; 2 Department of General Surgery, General Hospital of Central Theater Command, 627 Wuluo Road, Wu Chang District, Wuhan, 430070 China

**Keywords:** Colorectal carcinoma, Prediction model, Anastomotic leakage, Risk factor

## Abstract

**Background & Aims:**

Lymphocyte-C-reactive Protein Ratio (LCR) has been demonstrated as a promising new marker for predicting surgical and oncological outcomes in colorectal carcinoma (CRC). However, anastomotic leakage (AL) is also likely related to this inflammatory marker. Herein, we aimed to identify preoperative predictors of AL and build and develop a novel model able to identify patients at risk of developing AL.

**Methods:**

We collected 858 patients with CRC undergoing elective radical operation between 2007 and 2018 at a single center were retrospectively reviewed. We performed univariable and multivariable analyses and built a multivariable model that predicts AL based on preoperative factors. Propensity adjustment was used to correct the bias introduced by non-random matching of the LCR. The model's performance was evaluated by using the area under the receiver operator characteristic curves (AUROCs), decision curve analysis (DCA), Brier scores, D statistics, and R2 values.

**Results:**

Age, nutrition risk screening 2002 (NRS2002) score, tumor location and LCR, together with hemoglobin < 90 g/l, were independent predictors of AL. The models built on these variables showed good performance (internal validation: c-statistic = 0.851 (95%CI 0.803–0.965), Brier score = 0.049; temporal validation: c-statistic = 0.777 (95%CI 0.823–0.979), Brier score = 0.096). A regression equation to predict the AL was also established by multiple linear regression analysis: [Age(≥ 60 year) × 1.281] + [NRS2002(≥ 3) × 1.341] + [Tumor location(pt.) × 1.348]-[LCR(≤ 6000) × 1.593]-[Hemoglobin(< 90 g/L) × 1.589]-6.12.

**Conclusion:**

Preoperative LCR is an independent predictive factor for AL. A novel model combining LCR values, age, tumor location, and NRS2002 provided an excellent preoperative prediction of AL in patients with CRC. The nomogram can help clinical decision-making and support future research.

**Supplementary Information:**

The online version contains supplementary material available at 10.1186/s12893-022-01734-5.

## Background

Anastomotic leakage (AL) is a complex problem for patients with colorectal carcinoma (CRC) and the managing surgeon and is the most critical postoperative complication after surgical resection [1, 2]. AL harms patients' oncological, clinical and functional outcomes and is associated with high morbidity and mortality [3, 4]. The incidence of AL after colorectal surgery has been reported to be approximately 7–15% both nationally and internationally [5, 6].

In recent years, several prediction models and risk scores have been developed for colorectal AL, but most use a combination of multiple factors in the perioperative period (preoperative, intraoperative, and postoperative). In contrast, few studies have used only preoperative correlations to build predictive models for anastomotic fistulas. The results of these studies were, in addition, not validated in other cohorts to avoid overestimation of diagnostic performance for AL [7, 8].

It has been shown that inflammatory indicators (e.g., C-reactive protein, procalcitonin) are risk factors for postoperative AL [9, 10]. More current predictive models use a single inflammatory indicator, whereas a combination of inflammatory indicators will probably predict the incidence of AL more accurately. Lymphocyte-C-reactive protein (LCR) has been studied as a promising predictive marker of surgical and oncological outcomes in colorectal cancer [11], but further confirmation is needed as an independent predictor of AL.

This study aims to identify preoperative independent predictors of AL in patients undergoing radical resection for CRC and build a novel model able to identify patients at risk of AL.

## Methods

### Study design and data source

The reporting of this study conforms to STROBE guidelines [12]. Data were obtained from a retrospective database of CRC patients requiring surgical treatment at the Department of General Surgery, General Hospital of Central Theater Command between December 2007 and December 2018. Inclusion criteria were non-emergent procedures, first stage anastomosis, age ≥ 18 years and patients with a pathologically confirmed CRC diagnosis with imaging and colonoscopy suggestive of a single lesion and no distant metastases. Exclusion criteria were right semi colectomy, history of neoadjuvant therapy, documented evidence of intestinal obstruction and perforation before surgery, massive missing data regarding test indicators, transferred from an acute care hospital or outside the emergency department were ventilator-dependent. We used data from December 2007–December 2016 to develop a risk model and internal validation (n = 724) and data from January 2017–December 2018 (n = 134) for temporal validation. In addition, a propensity score matching was performed using the initial study cohort to investigate the correlation between LCR and AL.

Patient characteristics were obtained from the medical records system. We collected information on age, sex, body mass index (BMI), history of smoking, alcohol consumption, coronary heart disease, abdominal surgery, diabetes, hypertension, chronic obstructive pulmonary disease (COPD), hepatitis, kidney disease, hyperlipidemia, transfusion, and tumor location. Meanwhile, the Nutritional Risk Screening 2002 (NRS2002), American Society of Anesthesiologist (ASA) grade, and Eastern Cooperative Oncology Group (ECOG) score were also collected.

All patients had baseline fasting blood samples in the medical records system, obtained early in the morning of the day following admission. These blood samples were taken to the institution's testing department for routine blood, liver and kidney function, electrolytes, coagulation function, etc. The setup, layout, equipment, and facilities of the institution's clinical testing department comply with the Administrative Measures for Clinical Laboratories in Medical Institutions. Assay characteristics and coefficients of variation are available upon request.

Pre- and post-operative imaging includes CT or enhanced CT and MRI. When the preoperative tests are complete, a specialist surgical team, which usually includes 2–3 senior doctors, will have a preoperative discussion and confirm the final surgical approach. Depending on the location of the tumor, the surgical options include: 1. radical transverse colectomy + colon-colon end-to-side anastomosis 2. radical (extended) left hemicolectomy + colon-colon end-to-side anastomosis 3. radical sigmoidectomy + colon-rectum end-to-end anastomosis 4. anterior resection of rectum + colon-rectum end-to-end anastomosis or colon-anal tube end-to-end anastomosis. All patients had a postoperative abdominal (pelvic) drain placed. All operations were performed by experienced general surgeons (over five years of experience in colorectal tumor surgery) to ensure that the procedure was carried out to standard.

### Primary endpoint

Our primary endpoint was AL, which we defined and graded by reference to specific guidelines published by the International Study Group of Rectal Cancer (ISREC) in 2010 [13]. AL was defined as the defect of the intestinal wall integrity at the colon-colon, colon-rectal or colon-anal anastomotic site (including suture and staple lines of neorectal reservoirs) leading to a communication between the intra- and extraluminal compartments. A pelvic abscess close to the anastomosis is also considered as anastomotic leakage. AL was diagnosed by one or more following methods: postoperative CT scan; rigid or flexible colonoscopy; rectal touch; gaseous, fecal, or purulent discharge from a drainage tube; or relaparotomy. The degree of AL is divided into three categories: grade A requires no active therapeutic intervention; grade B requires active therapeutic intervention but manageable without relaparotomy; grade C requires relaparotomy. We defined all grades as outcomes in this study. Previous studies have used the same criteria to input outcome measures for prognostic modeling [14–16].

### Variable selection and conversion

We searched the PubMed, Embase, and Web of Science databases without language or time restrictions to retrieve relevant studies. In some retrospective cohort studies and published reviews, based on the results of these articles, we selected some predictor variables that were assessable early at hospital admission [11, 17–20], and these variables were combined with other preoperative factors added to the subsequent statistical analysis.

In our analysis, according to WHO standards, people with a physiological age of 60 years or older are considered elderly, so we classified age as ≥ 60 years and < 60 years. The NRS2002 score is commonly used to assess the nutritional status of patients [21]. A score of ≥ 3 often indicates a nutritional risk, and studies by Lee [22] and Kwag [23] have shown that patients with poor preoperative nutritional status are at increased risk for postoperative AL compared to the general population. We, therefore, classified the NRS2002 score as ≥ 3 and < 3. The lymphocyte to C-reactive protein ratio (LCR) has been shown to be a promising new marker for predicting surgical and oncological outcomes in colorectal cancer. A lower LCR (≤ 6000) could identify CRC patients who are at high risk for developing postoperative complications [11]. We have, therefore, divided the LCR into ≤ 6000 and > 6000 categories. Anemia is also a risk factor for AL after rectal surgery. In particular, moderate to severe anemia increases the incidence of postoperative AL [1, 2]. As 90 g/L is the cut-off marker for mild to moderate and severe anemia, we classified hemoglobin as < 90 g/L and ≥ 90 g/L. In patients who underwent one-stage colonic anastomosis, the tumors were located in the transverse colon, descending colon, sigmoid colon, and rectum. The descending colon and sigmoid colon were grouped in this study because of the similarity of the surgical approach.

### Model development

We used data from December 2007–December 2016 to develop a risk model (n = 724), performed an initial analysis and used multiple imputations with chained equations to replace missing values [24–27]. After multiple imputations were complete, univariate analysis was performed on 40 potential variables in the complete derivation data. All p values were two-sided, and risk factors with p values < 0.05 in the univariate analysis were included in a multivariate analysis. Multivariate logistic regression analysis was performed to identify independent risk factors. A backward method using the optimal subset function was used to filter out the best variables and develop a set of prediction models to predict the risk of anastomotic leakage.

### Model performance

We evaluated the overall predictive accuracy of the models with the R^2^ statistic [28]. Model discrimination was evaluated with the area under the receiver operator characteristic curves (AUROCs) and Harrell's C-statistic. Harrell's C-statistic [29] is a measure of discrimination similar to the AUROC but takes into account the censored nature of the data. Based on the literature, AUROC or Harrell's C-statistic greater than 0.7 was defined as a clinically useful prognostic score [30, 31]. Model calibration was evaluated graphically with calibration plots (plots of observed versus predicted outcomes) and statistically by computing the Brier score, which is generally considered a measure of the overall performance of the model but mainly a measure of the model calibration. The lower the Brier score is, the better the predictive calibration. Decision curve analysis (DCA) can circumvent the effects of false positives and false negatives in actual clinical work by quantifying the net benefits at different threshold probabilities [32]. DCA can explore a clinical judgment of the relative value of benefits and harms associated with the prediction model with LCR and without LCR.

### Model validation

Internal validation evaluates the stability of a prediction model to random changes in sample composition. Internal validation was performed by the enhanced bootstrap resampling technique, which used put-back resampling, and 100 resampling data points equal to the sample size of the model derivation cohort were reconstructed as the validation cohort. The model's performance in the original model derivation cohort and each validation cohort was calculated, and the difference between the two was calculated to obtain 100 high valuations. Finally, the mean high valuation was calculated, and the model performance in the original data was subtracted from the mean high valuation as the model performance in the internal validation. Data from January 2017–December 2018 (n = 134) for temporal validation. The predictor variables were transformed in the same way as the model derivation cohort. C-statistics, Brier score and DCA were used as indicators of model performance.

### Statistical analysis

Propensity score matching (PSM) can effectively reduce confounding effects and equalize the differences in confounding factors between groups, thus improving statistical efficiency. This study used PSM to match patients undergoing radical surgery for elective colorectal cancer and objectively evaluate the association between matched LCR and AL. We used calliper matching (CM) for 1:1 matching, with calliper value set at 0.05.

About the handling of missing data, in this study, there were no missing data for any variables (percentage of missing data in brackets), except BMI (10.62%), C-reactive protein (16.31%), uric acid (2.62%), APTT (0.96%), PT (0.96%), INR (0.96%), creatinine (1.13%), prealbumin (22.06%), albumin (3.86%), platelet count (0.68%) and hematocrit (0.68%). Variables with more than 50 percent missing data (e.g., procalcitonin, glycosylated hemoglobin, Serum IL-6, etc.) were not included in the study for this analysis. We filled in missing data using the technique of multiple imputations by chained equations, which samples imputed values from the posterior predictive distributions of missing data. We assumed data were missing at random. The imputation model was specified on all predictors, outcomes, and dummy variables for the study [33]. We performed 20 multiple imputations and finally obtained the complete dataset after imputation.

All categorical data were presented as number of cases and percentages, while continuous data were shown as mean ± standard deviation. As appropriate, for group comparisons of categorical and continuous variables, Chisquare test or Mann–Whitney test, T-test or Wilcoxon test and Jonckheere-Terpstra test were used. All p values were two-sided, and risk factors with p values < 0.05 in univariate analysis were included in a multivariate analysis. Multivariate logistic regression analysis was performed to identify independent risk factors, and a backward method was used to identify the useful combination of factors that could most precisely predict AL. A nomogram for anastomotic leakage was created based on the multivariate logistic regression model. The nomogram was constructed using the RMS package. DCA were constructed using the open-source “rmda” package. All analyses were performed with R software (version 4.0.3; R Foundation for Statistical Computing).

## Results

### Patient selection process and sample characteristics of the development and validation cohorts

**Fig. 1:**
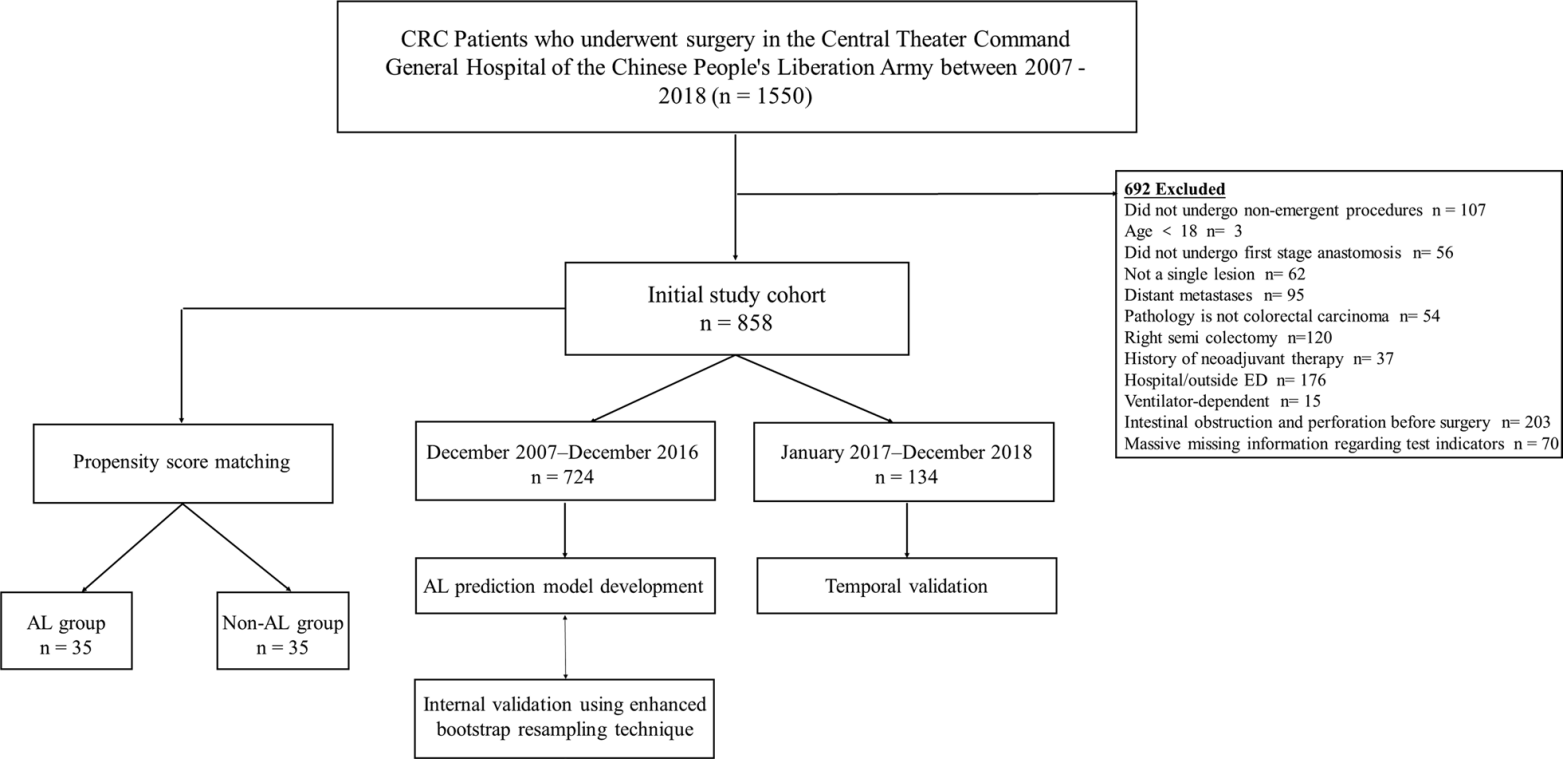
Flow diagram of patients selected for analysis

Between December 2007 and December 2018, 858 CRC cases met the eligibility criteria and were included in the initial study cohort. Excluded patients may have met more than 1 of the listed criteria. For subsequent analysis, the 724 and 134 patients were entered into the model development and temporal validation cohorts. The initial study cohort was included in the propensity-adjusted analyses (Fig. 1).

Table 1 shows baseline characteristics for anastomotic leakage in the internal and temporal validation cohorts. A total of 858 cases were included in the study, with 724 cases in the internal validation cohort and 134 cases in the temporal validation cohort. 45 patients were diagnosed with AL in the internal validation cohort (incidence rate of 6.21%) and 16 patients in the temporal validation cohort (incidence rate of 11.94%) after surgery. In a comparative analysis of the two cohorts (Additional file 1), we found a higher proportion of patients with rectal neoplasia (63.5% vs. 32.8%), a higher mean age (67.4 vs. 56.6), and more individuals with bowel preparation (99.9% vs. 91.0%) in the internal validation cohort. The temporal validation cohort had a higher number of patients with moderate to severe anemia (56.7% vs. 16.3%) and NRS2002 ≥ 3 (54.5% vs. 37.6%).

**Table 1:** Distribution of predictor variables in development and validation cohorts for model for prediction of AL. Continuous variables are expressed as mean ± standard deviation. Categorical variables are expressed as numbers (percentages) of patients

Factors	Internal validation cohort		Temporal validation cohort	
	Anastomotic Leakage n (%)	Non- leakage n (%)	leakage n (%)	Non- leakage N (%)
Age (years)	71.440 ± 7.524	65.760 ± 11.087	63.560 ± 11.260	55.70 ± 15.080
BMI (Kg/m.^2^)	23.175 ± 2.428	23.439 ± 11.326	22.823 ± 1.861	23.001 ± 3.568
Sex				
Male	33 (73.3)	393 (57.9)	11 (68.8)	71 (60.2)
Female	12 (26.7)	286 (42.1)	5 (31.2)	47 (39.8)
Smoking				
Yes	9 (20)	119 (17.5)	2 (12.5)	18 (15.3)
No	36 (80)	560 (82.5)	14 (87.5)	100 (84.7)
Alcohol				
Yes	8 (17.8)	78 (11.5)	2 (12.5)	13 (11)
No	37 (82.2)	601 (88.5)	14 (87.5)	105 (89)
Abdominal operation				
Yes	9 (20)	162 (23.9)	2 (12.5)	35 (29.7)
No	36 (80)	517 (76.1)	14 (87.5)	83 (70.3)
T2DM				
Yes	4 (8.9)	67 (9.9)	3 (18.8)	12 (10.2)
No	41 (91.1)	612 (90.1)	13 (81.2)	106 (89.8)
Cardiovascular disease				
Yes	2 (4.5)	70 (10.3)	1 (6.2)	17 (14.4)
No	43 (95.5)	609 (89.7)	15 (93.8)	101 (85.6)
Hypertension				
Yes	11 (24.4)	206 (30.3)	5 (31.2)	42(35.6)
No	34 (75.6)	473 (69.7)	11 (68.8)	76(64.4)
COPD				
Yes	4 (8.9)	41 (6)	0 (0)	15 (12.7)
No	41 (91.1)	638 (94)	16 (100)	103 (87.3)
Hepatitis				
Yes	1 (2.2)	23 (3.4)	0 (0)	6 (5.1)
No	44 (97.8)	656 (96.6)	16 (100)	112 (94.9)
Kidney disease				
Yes	1 (2.2)	16 (2.4)	0 (0)	0 (0)
No	44 (97.8)	663 (97.6)	16 (100)	118 (100)
Hyperlipidemia				
Yes	1 (2.2)	15 (2.2)	1 (6.2)	1 (0.8)
No	44 (97.8)	664 (97.8)	15 (93.8)	117 (99.2)
Transfusion history				
Yes	0 (0)	29 (4.3)	5 (31.2)	6 (5.1)
No	45 (100)	650 (95.7)	11 (68.8)	112 (94.9)
Bowel preparation				
Yes	45 (100)	678 (99.9)	11 (68.8)	111 (94.1)
No	0 (0)	1 (0.1)	5 (31.2)	7 (5.9)
Tumor location				
Rectum	39 (86.6)	421 (62)	9 (56.2)	35 (29.7)
Descending, sigmoid colon	4 (8.9)	119 (17.5)	5 (31.2)	38 (32.2)
Transverse colon	2 (4.5)	139 (20.5)	2 (12.5)	45 (38.1)
NRS2002				
≥3	32 (71.1)	240 (35.3)	10 (62.5)	63 (53.4)
<3	13 (28.9)	439 (64.7)	6 (37.5)	55 (46.6)
ASA score				
I	16 (35.6)	370 (54.5)	10 (62.5)	90 (76.3)
II	24 (53.3)	237 (34.9)	6 (37.5)	25 (21.2)
III	5 (11.1)	68 (10)	0 (0)	3 (2.5)
IV	0 (0)	4 (0.6)	0 (0)	0 (0)
ECOG score				
0	3 (6.7)	182 (26.8)	6 (37.5)	17 (14.4)
1	33 (73.3)	282 (41.5)	5 (31.2)	84 (71.2)
2	9 (20)	185 (27.2)	5 (31.2)	14 (11.9)
3	0 (0)	29 (4.3)	0 (0)	3 (2.5)
4	0 (0)	1 (0.1)	0 (0)	0 (0)
Hemoglobin (g/L)				
≥90	24 (53.3)	582 (85.7)	4 (25)	54 (45.8)
<90	21 (46.7)	97 (14.3)	12 (75)	64 (54.2)
LCR				
>6000	4 (8.9)	247 (36.4)	4 (25)	48 (40.7)
≤6000	41(91.1)	432 (63.6)	12 (75)	70 (59.3)
Total bilirubin (μmol/L)	12.655 ± 6.215	11.502 ± 6.979	15.519 ± 8.833	14.184 ± 15.831
Direct bilirubin (μmol/L)	3.157 ± 1.579	2.788 ± 1.389	4.219 ± 2.746	4.222 ± 9.001
ALT(IU/L)	16.910 ± 9.434	17.160 ± 12.514	13.000 ± 4.967	21.013 ± 18.674
AST(IU/L)	17.240 ± 7.496	18.570 ± 8.353	20.750 ± 6.758	22.208 ± 11.497
Prealbumin (g/L)	0.181 ± 0.061	0.275 ± 1.539	0.145 ± 0.045	0.205 ± 0.061
Albumin (g/L)	38.284 ± 3.727	38.836 ± 4.637	36.825 ± 4.732	39.014 ± 4.210
Urea (mmol/L)	4.983 ± 1.938	5.047 ± 2.196	4.763 ± 1.514	4.947 ± 1.745
Creatinine (μmol/L)	79.091 ± 14.861	76.610 ± 30.066	64.310 ± 15.890	64.680 ± 15.343
Uric acid (μmol/L)	296.969 ± 75.190	296.628 ± 88.906	311.38 ± 146.339	298.53 ± 82.366
White blood count (10^9^/L)	6.407 ± 2.556	6.185 ± 2.090	6.631 ± 2.578	5.873 ± 2.198
Neutrophil count (10^9^/L)	4.196 ± 2.471	3.885 ± 1.866	4.586 ± 2.213	3.621 ± 1.967
Lymphocyte count (10^9^/L)	1.521 ± 0.627	1.635 ± 0.616	1.314 ± 0.633	1.595 ± 0.555
Hematocrit (%)	36.993 ± 5.241	36.601 ± 13.687	31.956 ± 5.247	36.132 ± 5.454
Platelet count (10^9^/L)	207.089 ± 84.087	215.507 ± 74.742	283.940 ± 115.738	226.810 ± 71.798
APTT(s)	33.133 ± 4.601	32.785 ± 4.035	29.431 ± 4.649	33.218 ± 3.219
PT(s)	11.127 ± 0.936	11.130 ± 1.064	11.775 ± 0.921	11.350 ± 0.952
INR	0.990 ± 0.779	0.983 ± 0.113	1.086 ± 0.086	1.734 ± 7.453
C-reactive protein (ng/L)	7.726 ± 5.245	3.996 ± 7.342	8.549 ± 2.966	5.230 ± 4.536

*BMI* body mass index, *ASA* American Society of Anesthesiologists, *ECOG* Eastern Cooperative Oncology Group, *COPD* Chronic Obstructive Pulmonary Disease, *NRS2002* Nutritional Risk Screening 2002, *LCR* Lymphocyte-C-reactive protein Ratio, *ALT* alanine aminotransferase, *AST* aspartate aminotransferase, *T2DM* type 2 diabetes mellitus, *APTT* activated partial thromboplasin time, *PT* prothrombin time, *INR* international normalized ration

### Univariable and multivariable logistical regression

In the univariate analysis, 40 variables possibly associated with AL were studied. The results showed that Age (p < 0.001), Sex (p = 0.0041), Hemoglobin (p < 0.001), ASA score (p = 0.048), C-reactive protein (p = 0.001), ECOG score (p = 0.005), NRS2002 (p < 0.001), LCR (p < 0.001), and Tumor location (p = 0.003) were associated with the occurrence of anastomotic leakage. In the multivariate analysis, the following variables were confirmed to be independent risk factors for AL: Tumor location (p < 0.001), Age (p = 0.012), Sex (p = 0.041), Hemoglobin (p = 0.001), C-reactive protein (p = 0.040), LCR (p = 0.003) and NRS2002 (p = 0.006), (Table 2).

**Table 2:** Uni- and multivariate analysis of preoperative predictors of AL

		Development cohort			
		Univariate analysis		Multivariate analysis	
		OR (95% CI)	*p*	OR (95% CI)	*p*
Age (years)	(≥ 60/ < 60)	5.046 (1.785-14.263)	< 0.001	5.890 (1.701-27.623)	0.012
Sex	(Female/male)	0.501 (0.254-0.984)	0.041	0.420 (0.170-0.941)	0.044
BMI (Kg/m.^2^)		0.555 (0.294-1.047)	0.883		
Smoking	(Yes/No)	1.176 (0.552-2.507)	0.674		
Alcohol		1.666 (0.749-3.707)	0.207		
Abdominal operation	(Yes/No)	0.798 (0.376-1.691)	0.555		
T2DM	(Yes/No)	0.891 (0.310-2.565)	0.831		
Cardiovascular disease	(Yes/No)	0.405 (0.096-1.707)	0.203		
Hypertension	(Yes/No)	0.743 (0.369-1.495)	0.403		
COPD	(Yes/No)	1.518 (0.519-4.444)	0.443		
Hepatitis	(Yes/No)	0.648 (0.086-4.912)	0.672		
Kidney disease	(Yes/No)	0.942 (0.122-7.266)	0.954		
Hyperlipidemia	(Yes/No)	1.006 (0.130-7.792)	0.995		
Transfusion history	(Yes/No)	0.957 (0.942-0.973)	0.157		
Bowel preparation	(Yes/No)	0.999 (0.996-1.001)	0.797		
Hemoglobin (g/L)	(≥ 90/ < 90)	0.190 (0.102-0.355)	< 0.001	0.270 (0.120-0.643)	0.001
Tumor location	Transverse colon descending, sigmoid colon rectum	0.291 (0.009-9.958)	0.003	3.940 (2.100-8.610)	< 0.001
NRS2002	(≥ 3/ < 3)	4.503 (2.319-8.743)	< 0.001	3.240 (1.441-7.741)	0.006
LCR	(> 6000/ ≤ 6000)	0.171 (0.060-0.482)	< 0.001	0.180 (0.053-0.530)	0.003
ASA score	(I/II/III/IV)	0.386 (0.002-63.381)	0.048	1.260 (0.681-2.310)	0.510
ECOG score	(0/1/2/3/4)	16.556 (0.610-49.231)	0.005	0.862 (0.490-1.510)	0.610
Total bilirubin(μmol/L)		1.007 (0.863-1.175)	0.280		
Direct bilirubin(μmol/L)		1.537 (0.328-7.190)	0.088		
ALT (IU/L)		0.930 (0.684-1.265)	0.895		
AST (IU/L)		0.879 (0.617-1.254)	0.298		
Prealbumin (g/L)		0.224 (0.004-12.783)	0.684		
Albumin (g/L)		0.956 (0.611-1.497)	0.435		
Urea (mmol/L)		0.329 (0.060-1.787)	0.848		
Creatinine (μmol/L)		0.915 (0.776-1.080)	0.583		
Uric acid (μmol/L)		1.002 (0.972-1.033)	0.980		
White blood count (10^9^/L)		0.466 (0.084-1.213)	0.497		
Neutrophil count (10^9^/L)		0.684 (0.103-1.641)	0.290		
Lymphocyte count (10^9^/L)		1.445 (0.227-10.360)	0.229		
Hematocrit (%)		1.048 (0.937-1.172)	0.848		
Platelet count (10^9^/L)		0.973 (0.933-1.015)	0.468		
APTT(s)		1.067 (0.689-1.651)	0.578		
PT(s)		3.085 (0.188-50.598)	0.986		
INR		6.011 (1.308-15.855)	0.646		
C-reactive protein(ng/L)		2.364 (0.985-5.215)	0.001	1.031 (0.990-1.060)	0.040

*BMI* body mass index, *ASA* American Society of Anesthesiologists, *ECOG* Eastern Cooperative Oncology Group, *COPD* Chronic Obstructive Pulmonary Disease, *NRS2002* Nutritional Risk Screening 2002, *LCR* Lymphocyte-C-reactive protein Ratio, *ALT* alanine aminotransferase, *AST* aspartate aminotransferase, *T2DM* type 2 diabetes mellitus, *APTT* activated partial thromboplasin time, *PT* prothrombin time, *INR* international normalized ration

The AL independent risk factors from the multivariate analysis were used to obtain the final variables in the prediction model by backward method: Tumor location (p < 0.001), NRS2002 (p < 0.001), Hemoglobin(g/L) (p < 0.001), LCR (p = 0.002), Age (years) (p = 0.015).

### Correlation analysis of LCR and AL after PSM

Age, gender, hemoglobin, tumor location, NRS2002, ASA score, ECOG score were unevenly distributed between the two groups before PSM, and the difference was statistically significant. (p < 0.05). We match variables other than LCR using PSM. After PSM, a total of 35 pairs were successfully matched, and the distribution of age, sex, hemoglobin, tumor location, NRS2002, ASA score, and ECOG score was balanced between the two groups after matching, and the difference was not statistically significant (p > 0.05). At this time, the baseline characteristics of the two groups were balanced and comparable, and the difference in LCR between the two groups was compared using the chi-square test, which revealed a higher number of low LCR in the anastomotic fistula group and a statistically significant difference (p < 0. 01), (Table 3).

**Table 3:** Comparison of baseline characteristics after PSM in two groups

Variables		Anastomotic leakage n = 35	Non-anastomotic leakage Statistical values n = 35		p
Age	(< 60/ ≥ 60)	5/30	7/28	1.867	0.172
Sex	(Male/female)	24/11	25/10	0.068	0.794
BMI (Kg/m^2^)		23.6 ± 2.3	22.9 ± 2.5	0.086	0.700
Smoking	(No/Yes)	28/7	26/9	0.324	0.569
Alcohol	(No/Yes)	30/5	32/3	0.565	0.452
Abdominal operation	(No/Yes)	27/8	27/8	0	> 0.05
T2DM	(No/Yes)	33/2	31/4	0.729	0.393
Cardiovascular disease	(No/Yes)	34/1	33/2	0.348	0.555
Hypertension	(No/Yes)	25/10	26/9	0.072	0.788
COPD	(No/Yes)	31/4	33/2	0.729	0.393
Hepatitis	(No/Yes)	34/1	34/1	0	> 0.05
Kidney disease	(No/Yes)	34/1	34/1	0	> 0.05
Hyperlipidemia	(No/Yes)	35/0	34/1	1.014	0.314
Transfusion history	(No/Yes)	35/0	35/0	0	> 0.05
Bowel preparation	(No/Yes)	0/35	0/35	0	> 0.05
Hemoglobin (g/L)	(< 90/ ≥ 90)	15/20	14/21	0.059	0.808
Tumor location	Transverse colon descending, sigmoid colon rectum	2/4/29	0/2/33	2.925	0.232
NRS2002	(< 3/ ≥ 3)	9/26	10/25	0.072	0.788
LCR	(≤ 6000/ > 6000)	32/3	11/24	26.589	< 0.01
ASA score	(I/II/III/IV)	14/16/5/0	16/16/2/1	2.419	0.490
ECOG score	(0/1/2/3/4)	2/26/7/0	8/20/5/2	6.716	0.082
Total bilirubin (μmol/L)		11.1 ± 5.2	13.1 ± 6.7	0.002	0.194
Direct bilirubin (μmol/L)		3.3 ± 1.6	2.7 ± 1.4	0.356	0.127
ALT (IU/L)		17.6 ± 10.1	15.5 ± 9.5	0.153	0.364
AST (IU/L)		18.0 ± 8.2	18.3 ± 8.5	0.083	0.887
Prealbumin (g/L)		0.18 ± 0.05	0.21 ± 0.04	0.057	0.105
Albumin (g/L)		37.9 ± 4,0	38.1 ± 4.4	0.461	0.898
Urea (mmol/L)		4.77 ± 2.0	5.5 ± 4.0	0.249	0.284
Creatinine (μmol/L)		77.9 ± 15.9	87.1 ± 65.9	1.444	0.424
Uric acid (μmol/L)		295.0 ± 78.9	289.1 ± 76.3	0.055	0.750
White blood count(10^9^/L)		6.6 ± 2.8	5.7 ± 1.4	5.966	0.080
Neutrophil count (10^9^/L)		4.3 ± 2.7	3.4 ± 1.3	4.565	0.085
Lymphocyte count (10^9^/L)		1.5 ± 0.7	1.5 ± 0.6	0.995	0.989
Hematocrit (%)		34.5 ± 5.1	32.6 ± 6.1	1.935	0.145
Platelet count (10^9^/L)		208.7 ± 93.4	212.9 ± 77.4	0.047	0.835
APTT(s)		32.6 ± 4.5	33.6 ± 4.6	0.154	0.361
PT(s)		11.3 ± 0.9	11.3 ± 0.8	1.019	0.937
INR		0.99 ± 0.07	0.98 ± 0.07	0.037	0.619

*BMI* body mass index, *ASA* American Society of Anesthesiologists, *ECOG* Eastern Cooperative Oncology Group, *COPD* Chronic Obstructive Pulmonary Disease, *NRS2002* Nutritional Risk Screening 2002, *LCR* Lymphocyte-C-reactive protein Ratio, *ALT* alanine aminotransferase, *AST* aspartate aminotransferase, *T2DM* type 2 diabetes mellitus, *APTT* activated partial thromboplasin time, *PT* prothrombin time, *INR* international normalized ration

### Analysis of risk factors associated with AL severity

We used a non-parametric test (Jonckheere-Terpstra test) to determine whether the severity of AL (grades A, B, and C) was correlated with the variables. The results showed that LCR, Hemoglobin, Prealbumin, and Creatinine were associated with the severity of AL (Additional file 2). The variables were judged to be more strongly correlated with which grade based on mean rank. We further stratified analyzed the above variables in grade A vs. B, A vs. C, B vs. C, AB vs. C, and A vs. BC. The results showed statistically significant differences in LCR, Hemoglobin, Prealbumin, and Creatinine in A vs. B and A vs. BC, and no statistically significant differences in A vs. C, B vs. C, and AB vs. C (Additional file 2).

### Internal and temporal validation

**Fig. 2:**
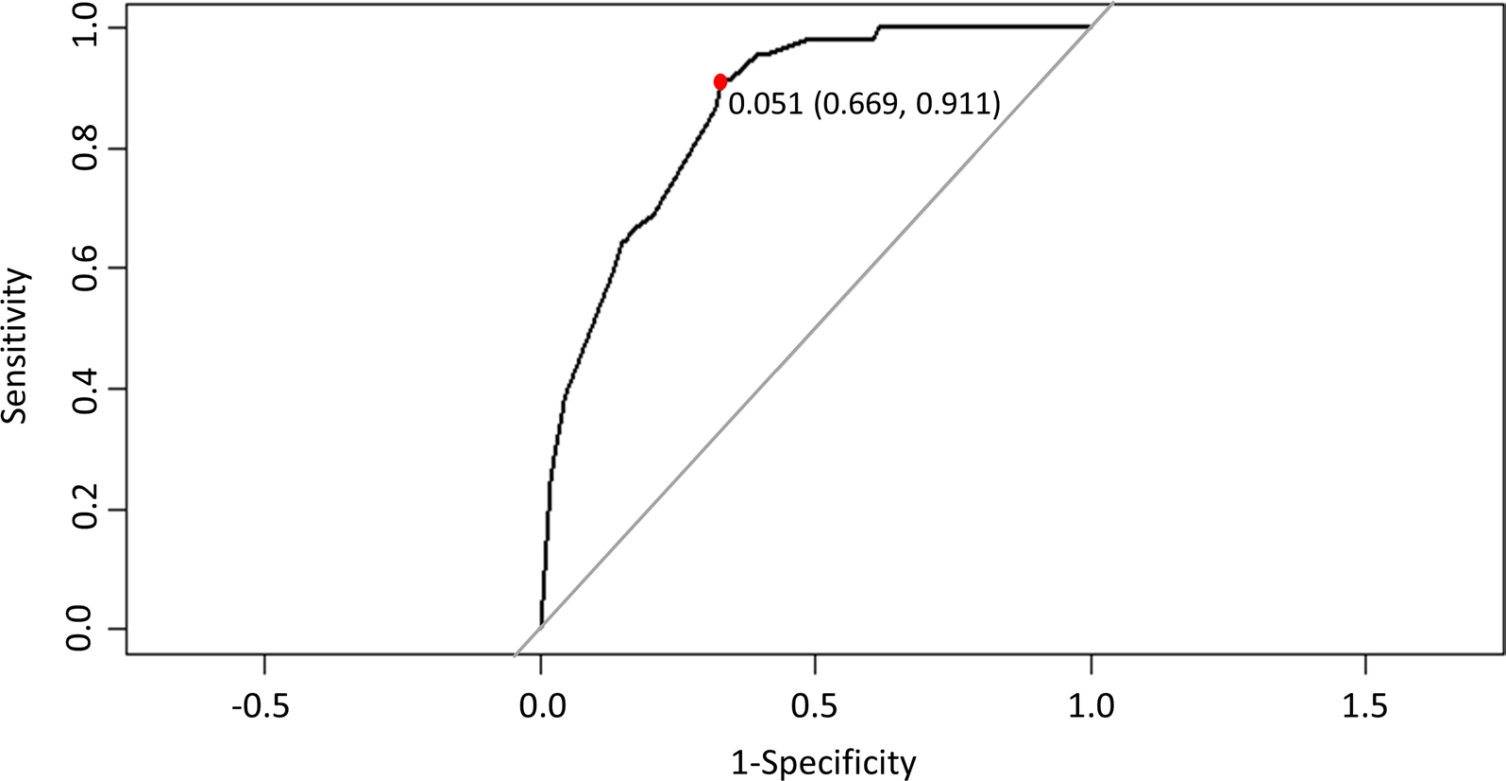
Receiver operating characteristic curve for the prediction model Area under the curve was 0.871 (95% confidence interval 0.814–0.904)

**Fig. 3:**
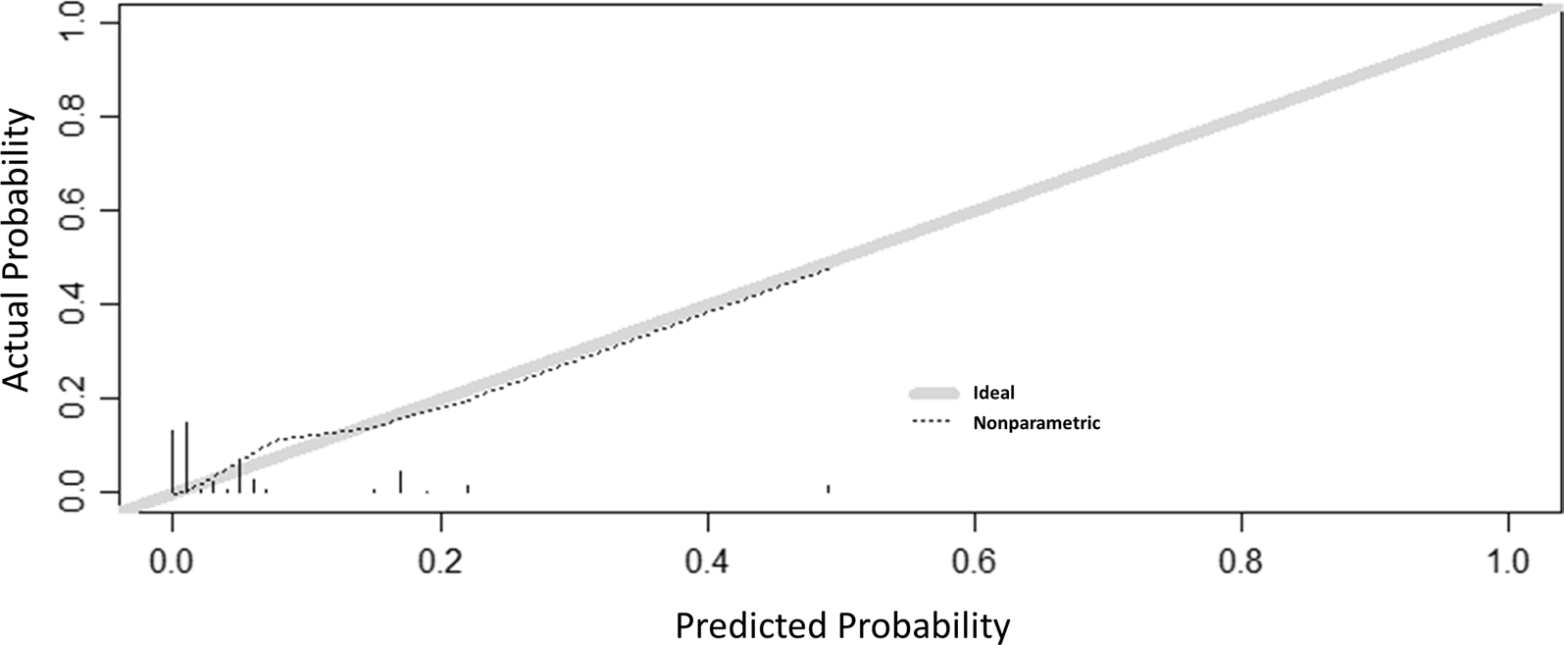
Calibration of the model for anastomotic leakage The x-axis shows the predicted probability of anastomotic leakage, and the y-axis shows the observed probability of anastomotic leakage

The predictive performance of the original model derivation cohort was assessed by ROC analysis according to binary outcomes, as shown in Fig. 2. From the ROC analysis, the area under the curve was 0.871 (95% CI, 0.814–0.904; p < 0.001). We also obtained their sensitivity and specificity by ROC analysis. When the cut-off point was 0.051, maximum sensitivity and specificity were obtained (0.669 and 0.911, respectively). The calibration plot is shown in Fig. 3, and the Brier score was 0.047. Using enhanced bootstrap resampling for internal validation, we obtained an AUROC of 0.851 (95% CI, 0.803–0.965) and a Brier score of 0.049, indicating that the model had good discrimination and calibration.

In this dataset, R^2^ values were 0.059. The AUROC was 0.777 (95% CI, 0.823–0.979), and the Brier score was 0.096, indicating that the model had good discrimination and calibration. The calibration intercept and slope for 0.161 and 0.674, respectively. Table 4 shows the performance of each algorithm in the external validation and internal validation cohorts.

**Table 4:** Mean (95% confidence interval) performance of model in the internal and temporal validation cohort

Statistic	Internal validation	Temporal validation
D statistic*	0.111 (0.110-0.113)	0.024 (0.022-0.026)
Harrell's C*	0.851 (0.803-0.965)	0.777 (0.823-0.979)
R^2^(%)†	0.302 (0.298-0.306)	0.159 (0.155-0.162)
Brier‡	0.049 (0.047-0.051)	0.096 (0.094-0.098)

*A measure of discrimination. Higher values indicate better discrimination

^†^Measures explained the degree of fit between the cohort and the fitted model. Higher values indicate better fit

^‡^A measure of calibration. Lower values indicate better calibration

### Decision curve analysis to the judgment of the relative value of LCR

**Fig. 4:**
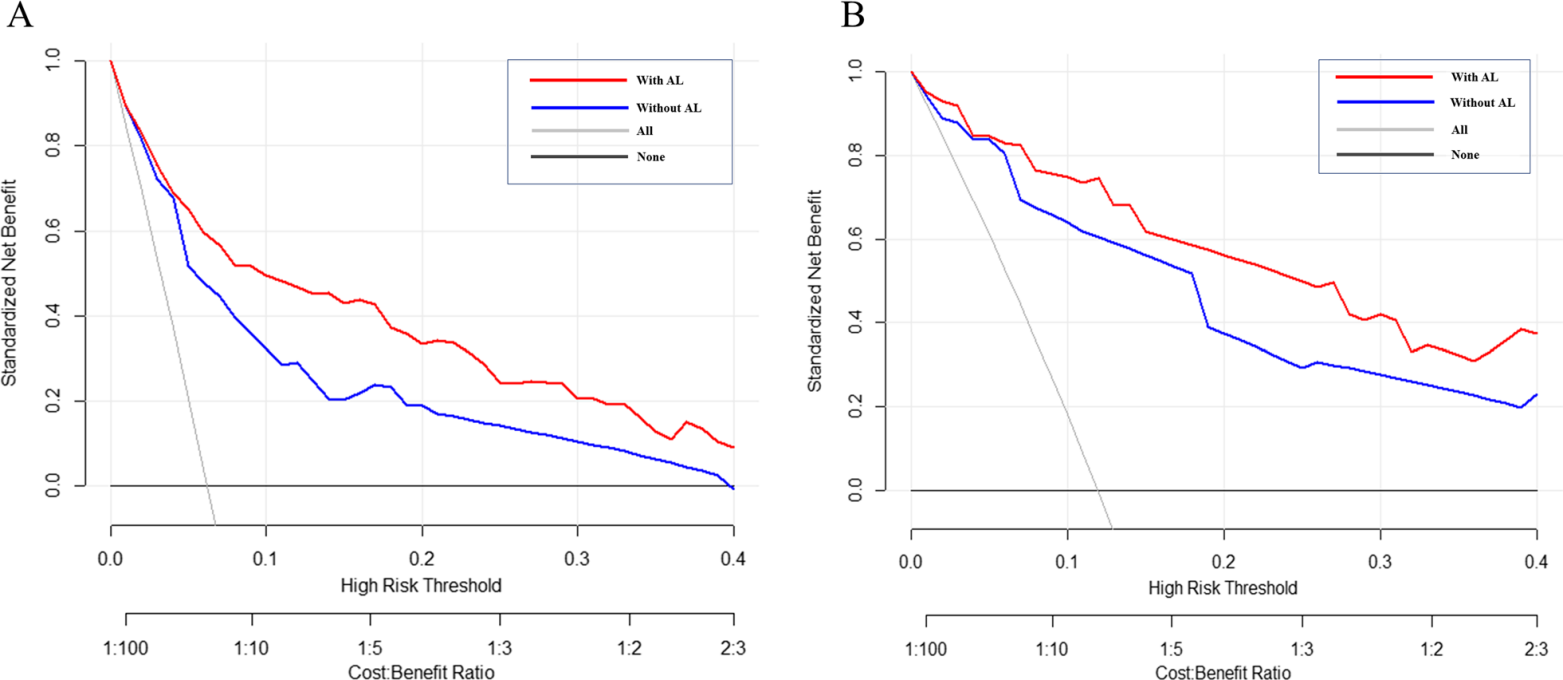
Decision curve analysis of the prediction model with and without LCR in internal **A** and temporal **B** cohort. The horizontal coordinate of the graph is the threshold probability, and the vertical coordinate is the net benefit

We applied DCA to explore the relative judgment of the model with and without LCR on clinical predictive value. We performed DCA in both the internal and temporal validation cohorts (Fig. 4) and corrected for net benefit using the standardized setting. The results show that in the internal validation cohort, within a threshold of 0.1 to 0.4, the net benefit is higher for models with LCR than for models without LCR. Similar results were shown in the temporal validation cohort, with higher net benefit rates for models with LCR than for models without LCR within a threshold range of 0.15 to 0.4.

### Model presentation

**Fig. 5:**
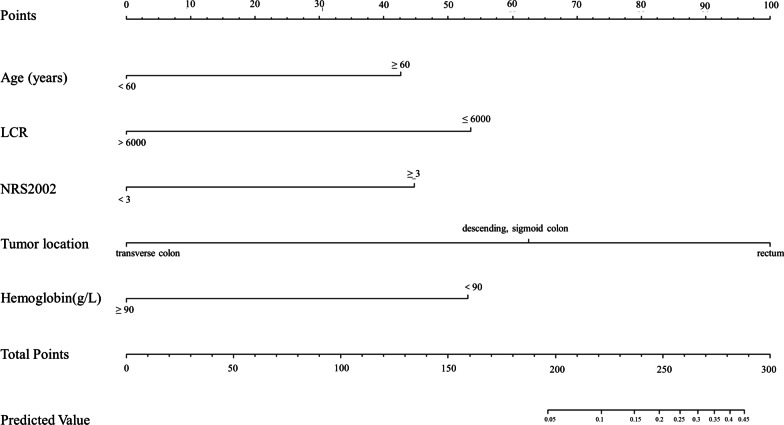
Nomogram predicting the probability of AL. To estimate the probability of AL, mark patient values at each axis, draw a straight line perpendicular to the point axis, and sum the points for all variables. Next, mark the sum on the total point axis and draw a straight line perpendicular to the probability axis

We developed a nomogram that included the following variables: tumor location, NRS2002, hemoglobin, age, and LCR (Fig. 5). Moreover, to predict AL, a regression equation was established by multiple linear regression analysis: [Age(≥ 60 year) × 1.281] + [NRS2002(≥ 3) × 1.341] + [Tumor location(pt.) × 1.348]-[LCR(≤ 6000) × 1.593]-[Hemoglobin(< 90 g/L) × 1.589]-6.12.

## Discussion

This study shows that LCR can be an independent predictor of AL and a predictive model for postoperative AL in colorectal cancer was developed in combination with preoperative related risk factors. The model was validated in the internal and temporal cohorts. We also created the nomogram as a reference for clinicians and surgeons to help in clinical decision-making. Previous reports have described many potential risk factors for AL after colorectal cancer [17, 18, 20, 34]. These risk factors include a wide range of factors in the perioperative period, and those predictive of preoperative factors alone have not yet been reported. In addition, most studies were not validated in a temporal or external cohort. Age, sex, tumor location, hemoglobin, ASA score, C-reactive protein, ECOG score, LCR, and NRS2002 were identified as potential preoperative risk factors for AL in research. Among these, we identified the most relevant factors: tumor location, NRS2002, hemoglobin, LCR, and age. Our prediction models for AL performed well in the internal and temporal validation cohorts. The models had good discrimination and calibration. The predictor items are assessable before surgery.

Several studies have shown that different types of inflammatory factors have the potential to be used as prognostic indicators in determining human malignancies [35]. However, it is still unclear which preoperative inflammatory index is better for predicting the risk of postoperative anastomotic leak in patients with colorectal cancer. In this study, we added LCR to the filter of predictor variables, which was not present in previous models predicting anastomotic fistula. Meanwhile, we not only developed a preoperative predictive model based on LCR but also explored some essential findings of LCR through multifaceted statistical analysis. Firstly, we found by logistical regression that LCR may be a more reliable indicator of postoperative AL in CRC patients and an independent risk factor compared to other inflammatory indicators (e.g., neutrophils, lymphocyte count, etc.). Secondly, our overall and stratified analysis of AL severity suggested a potential association between LCR and AL severity. Finally, we also performed DCA analyses in both internal and temporal validation cohorts, demonstrating the significant predictive value of LCR in the model.

Thus, preoperative LCR assessment of patients, combined with CRC-related antimicrobial use guidelines [36], may help surgeons decide which antibiotics to utilize prophylactically and whether to use them for a duration longer than 24 h to prevent postoperative AL and other infectious complications in colorectal cancer patients. At the same time, assessment of predictive models can identify groups at high risk of postoperative AL (especially low LCR), who may require closer postoperative monitoring and actively supporting therapy to improve the development of postoperative complications in patients with CRC. Although our study showed an association of low LCR with postoperative AL as a potential independent predictor, it also performed better than other inflammatory factors. However, this study is a single-center retrospective study, and further investigation through multi-center studies with larger samples and long-term tumor follow-up results is still needed.

The NRS2002 scale is commonly used to assess patients' nutritional status comprehensively. Previous results have shown that the NRS2002 score is associated with postoperative complications in patients [37, 38]. Patients with poor preoperative nutritional status are at increased risk of postoperative anastomotic leakage, which may be due to the prolonged healing time of the anastomosis. Therefore, the preoperative nutritional risk assessment should be routinely performed in CRC patients, and patients with poor nutritional status should be given nutritional support before elective surgery to reduce the incidence of postoperative anastomotic leak. Anemia is also a risk factor for anastomotic leakage after CRC surgery. It is estimated that more than 40% of CRC patients are anemia, and more than 25% are moderate to severely anemia [39, 40]. Therefore, preoperative correction of anemia, exceptionally moderate to severe anemia, should be attempted to reduce the incidence of preoperative anastomotic leak.

Our study found that most anastomotic leakage occurred in the colon-rectal anastomosis, consistent with the clinical presentation. The main reasons for this may be related to the narrow operating space at the pelvis and the fact that the length of the descending colon varies from patient to patient, resulting in a shorter remaining colon after removal of the intestinal canal where the tumor is located, leading to relatively greater anastomotic tension. Also, the probability of transverse colonic anastomotic fistula was lower in this study compared to the descending and sigmoid colon, which is inconsistent with many studies. The ascending and descending colon are intercolonic organs, which are relatively fixed in position and prone to excessive tension during anastomosis. Taken together, all these variables performed well in our model to predict AL.

The nomogram could provide the surgeon with the probability of AL after one-stage anastomosis for colorectal cancer. The nomogram is composed of preoperative risk factors. Predictor variables are readily available, even in small hospitals. When the nomogram identifies patients with a higher probability of AL, they should be monitored carefully during the postoperative period. It might be helpful for them to delay the resumption of oral intake or remove drainage tubes [15]. The usefulness of nomograms for AL has been reported [41–43], with the advantage of accurately predicting risk in patients. Even though the performance of such a nomogram was internally well-demonstrated through bootstrapping, the use of an external validation set is generally recommended.

There was a statistically significant difference in AL rates between the internal validation and temporal validation cohorts in this study (6.2% vs 11.9%). In this regard, we performed a comparative analysis of the two cohorts (Additional files 4 and 5). We found that the baseline data for the two cohorts differed in some variables and that the baselines were not evenly comparable. The proportion of patients with hemoglobin < 90 and NRS2002 ≥ 3 was more significant in the temporal validation cohort than in the internal validation cohort (p < 0.001). These two variables were also the two variables that accounted for a greater proportion of predictions in the model, which may have contributed to the differences in AL rates between the two cohorts. In addition, the mean age of the patients and the proportion of rectal tumors were higher in the internal validation than in the temporal validation cohort, which may be responsible for the lower AUROC in the temporal validation cohort (0.777) compared to the internal validation cohort AUROC (0.851). However, the model still has reliable discrimination and calibration in the analysis of this study. The next step is to validate the model with multi-center data to demonstrate its generalisability further.

There are other potential limitations to our study. Due to data collection in a single-center retrospective database, recall bias is inevitable. In addition, some potential predictor variables were not included in the model due to large differences in certain variables or large amounts of missing data in laboratory tests performed after admission, and the forced application of statistical interpolation methods would have led to confusion in the data (e.g., procalcitonin and glycated hemoglobin). There is the possibility of updating the models as more reliable predictors of outcome are identified.

This study focuses on the effect of preoperative factors on AL and develops a predictive model for this. Therefore, there is no mention of its surgical and postoperative factors. At the same time, we hope to balance some relevant confounding factors better to explore the association between preoperative factors and AL. The patients we included in this study were all those who underwent one-stage large intestine-large intestine anastomosis (colon-colon, colon-rectum, and colon-anal tube) and did not include patients who underwent radical right hemicolectomy. Firstly, these patients most underwent small intestine—large intestine anastomosis, which is theoretically less likely to develop an AL due to the relatively free small bowel and its lower anastomotic tension than large bowel-colon anastomosis. Secondly, there are differences in the physiological characteristics of the small intestine—large intestine anastomosis versus large intestine-large intestine anastomosis. Therefore, including these patients for analysis and predictive model construction may potentially bias and affect the model's performance.

Rather than excluding all patients with missing data from the analysis, we used data imputation to reduce the impact of data loss. The proportion of missing data also differed between the development and validation sets. Nevertheless, multiple imputations are now widely accepted and increasingly used after theoretical and empirical evidence that the technique is superior to traditional complete case analysis [33]. The ideal situation is, of course, that all patient data are fully available. Finally, our AL prediction models could be helpful for research purposes and the advancement of the design and analysis of some clinical trials in AL. For example, they could provide more evidence to improve some preoperative indicators before surgery for patients undergoing elective surgery to reduce the risk of postoperative complications.

## Conclusion

In conclusion, LCR, together with available clinical and biochemical data, may provide an excellent preoperative prediction of AL in patients with CRC. Preoperative assessment of the LCR may help the surgeon individualize treatment more effectively during the perioperative period. External validation of the current model will be performed in future studies.

## Supplementary Information

**Additional file 1: Table S1.** Comparison of the distribution of variables in the internal and temporal validation cohorts.

**Additional file 2: Table S2-1.** Correlation analysis of AL severity (Jonckheere-Terpstra Test), **Table S2-2**. Correlation of AL severity with LCR and other factors.

**Additional file 3: Table S3.** Distribution of predictor variables in overall cohorts for model for prediction of AL. Continuous variables are expressed as mean±standard deviation. Categorical variables are expressed as numbers (percentages) of patients.

**Additional file 4: Table S4.** Uni- and multivariate analysis of preoperative predictors of AL (overall analysis).

**Additional file 5: Table S5.** Uni- and multivariate analysis of preoperative predictors of AL (right hemi colectomy).

## Data Availability

The datasets generated and/or analyzed during the current study are not publicly available due to the closed management of patient information in military hospitals and at the same time, the data are not publically available as they are pseudonymised medical records. But are available from the corresponding author on reasonable request.
