# Supplementary material for: A preoperative prediction model based on Lymphocyte-C-reactive protein ratio predicts postoperative anastomotic leakage in patients with colorectal carcinoma: a retrospective study
Source: BMC Surg. 2022 Jul 23;22:283. doi: 10.1186/s12893-022-01734-5 (PMC9308913; doi:10.1186/s12893-022-01734-5)
Supplement: Supplementary file 1 — Additional file 1: Table S1. Comparison of the distribution of variables in the internal and temporal validation cohorts. [file 12893_2022_1734_MOESM1_ESM.docx]

Additional file 1: Table S1. Comparison of the distribution of variables in the internal and temporal validation cohorts.

| **Factors** | **Internal validation cohort** | **Temporal validation cohort** | **p value** |
| --- | --- | --- | --- |
|  | **n (%)** | **n (%)** |  |
| No. of patients | 724 (100.0) | 134 (100.0) |  |
| Anastomotic leakage |  |  | <0.001 |
| Yes | 45 (6.2) | 16 (11.9) |  |
| No | 679 (93.8) | 118 (88.1) |  |
| Age (years) | 67.4±10.8 | 56.6±14.9 | 0.014 |
| BMI (Kg/m^2^) | 21.9±10.1 | 23.0±3.4 | 0.064 |
| Sex |  |  | 0.610 |
| Male | 426 (58.8) | 82 (61.2) |  |
| Female | 298 (41.2) | 52 (38.8) |  |
| Smoking |  |  | 0.438 |
| Yes | 128 (17.7) | 20 (14.9) |  |
| No | 596 (82.3) | 114 (85.1) |  |
| Alcohol |  |  | 0.821 |
| Yes | 86 (11.9) | 15 (11.2) |  |
| No | 638 (88.1) | 119 (88.8) |  |
| Abdominal operation |  |  | 0.039 |
| Yes | 171 (23.6) | 37 (27.6) |  |
| No | 553 (76.4) | 97 (72.4) |  |
| T2DM |  |  | 0.623 |
| Yes | 71 (9.8) | 15 (11.2) |  |
| No | 653 (90.2) | 119 (88.8) |  |
| Cardiovascular disease |  |  | 0.226 |
| Yes | 72 (9.9) | 18 (13.4) |  |
| No | 652 (90.1) | 116 (86.6) |  |
| Hypertension |  |  | 0.240 |
| Yes | 217 (30.0) | 47 (35.1) |  |
| No | 507 (70.0) | 87 (64.9) |  |
| COPD |  |  | 0.051 |
| Yes | 45 (6.2) | 15 (11.2) |  |
| No | 679 (93.8) | 119 (88.8) |  |
| Hepatitis |  |  | 0.501 |
| Yes | 24 (3.3) | 6 (4.5) |  |
| No | 700 (96.7) | 128 (95.5) |  |
| Kidney disease |  |  | 0.073 |
| Yes | 17 (2.3) | 0 |  |
| No | 707 (97.7) | 134 (100.0) |  |
| Hyperlipidemia |  |  | 0.595 |
| Yes | 16 (2.2) | 2 (1.5) |  |
| No | 708 (97.8) | 132 (98.5) |  |
| Transfusion history |  |  | 0.071 |
| Yes | 29 (4.0) | 11 (8.2) |  |
| No | 695 (96.0) | 123 (91.8) |  |
| Bowel preparation |  |  | <0.001 |
| Yes | 723 (99.9) | 122 (91.0) |  |
| No | 1 (0.1) | 12 (9.0) |  |
| Tumor location |  |  | <0.001 |
| rectum | 460 (63.5) | 44 (32.8) |  |
| descending, sigmoid colon | 123 (17.0) | 43 (32.1) |  |
| transverse colon | 141 (19.5) | 47 (35.1) |  |
| NRS2002 |  |  | <0.001 |
| ≧3 | 272 (37.6) | 73 (54.5) |  |
| ﹤3 | 452 (62.4) | 61 (45.5) |  |
| ASA score |  |  | <0.001 |
| Ⅰ | 386 (53.3) | 100 (74.6) |  |
| Ⅱ | 261 (36.0) | 31 (23.1) |  |
| Ⅲ | 73 (10.1) | 3 (2.2) |  |
| Ⅳ | 4 (0.6) | 0 |  |
| ECOG score |  |  | <0.001 |
| 0 | 185 (25.6) | 23 (17.2) |  |
| 1 | 315 (43.5) | 89 (66.4) |  |
| 2 | 194 (26.8) | 19 (14.2) |  |
| 3 | 29 (4.0) | 3 (2.2) |  |
| 4 | 1 (0.1) | 0 |  |
| Hemoglobin (g/L) |  |  | <0.001 |
| ≧90 | 606 (83.7) | 58 (43.3) |  |
| ﹤90 | 118 (16.3) | 76 (56.7) |  |
| LCR |  |  | 0.357 |
| ﹥6000 | 251 (34.7) | 52 (38.8) |  |
| ≦6000 | 473 (65.3) | 82 (61.2) |  |
| Total bilirubin (μmol/L) | 11.6±6.9 | 14.3±15.1 | 0.040 |
| Direct bilirubin (μmol/L) | 2.8±1.4 | 4.2±8.5 | 0.057 |
| ALT(IU/L) | 17.1±12.3 | 20.1±17.8 | 0.071 |
| AST(IU/L) | 18.5±8.3 | 22.0±11.0 | 0.091 |
| Prealbumin(g/L) | 0.3±1.5 | 0.2±0.1 | 0.579 |
| Albumin(g/L) | 38.8±4.6 | 38.8±4.3 | 0.908 |
| Urea(mmol/L) | 5.0±2.2 | 4.9±1.7 | 0.554 |
| Creatinine(μmol/L) | 76.8±29.3 | 64.6±15.3 | 0.036 |
| Uric acid(μmol/L) | 296.6±88.1 | 300.1±91.7 | 0.682 |
| White blood count (10⁹/L) | 6.2±2.1 | 6.0±2.2 | 0.244 |
| Neutrophil count (10⁹/L) | 3.9±1.9 | 3.7±2.0 | 0.351 |
| Lymphocyte count (10⁹/L) | 1.6±0.6 | 1.6±0.6 | 0.247 |
| Hematocrit(%) | 36.6±13.3 | 35.6±5.6 | 0.397 |
| Platelet count (10⁹/L) | 215.0±75.3 | 233.6±79.9 | 0.033 |
| APTT(s) | 32.8±4.1 | 32.8±3.6 | 0.914 |
| PT(s) | 11.1±1.1 | 11.4±1.0 | 0.034 |
| INR | 0.9±0.1 | 1.7±0.7 | 0.267 |
| C-reactive protein (ng/L) | 4.2±7.3 | 5.6±4.5 | 0.253 |

Abbreviations: BMI, body mass index; ASA, American Society of Anesthesiologists; ECOG, Eastern Cooperative Oncology Group; COPD, Chronic Obstructive Pulmonary Disease; NRS2002, Nutritional Risk Screening 2002; LCR, Lymphocyte-C-reactive protein Ratio; ALT, alanine aminotransferase; AST, aspartate aminotransferase; T2DM, type 2 diabetes mellitus; APTT, activated partial thromboplasin time; PT, prothrombin time; INR, international normalized ration.
