# Supplementary material for: A preoperative prediction model based on Lymphocyte-C-reactive protein ratio predicts postoperative anastomotic leakage in patients with colorectal carcinoma: a retrospective study
Source: BMC Surg. 2022 Jul 23;22:283. doi: 10.1186/s12893-022-01734-5 (PMC9308913; doi:10.1186/s12893-022-01734-5)
Supplement: Supplementary file 2 — Additional file 2: Table S2-1. Correlation analysis of AL severity (Jonckheere-Terpstra Test), Table S2-2. Correlation of AL severity with LCR and other factors. [file 12893_2022_1734_MOESM2_ESM.docx]

Supplement 2

Table 2-1. Correlation analysis of AL severity (Jonckheere-Terpstra Test).

| Factors | AL severity(n) | | Mean Rank | statistics | *p* |
| --- | --- | --- | --- | --- | --- |
| Age | | A (23)  B (20)  C (2) | 23.20  22.50  25.00 | 0.045 | 0.964 |
| Sex | | A (23)  B (20)  C (2) | 25.10  20.75  17.00 | -1.338 | 0.181 |
| BMI (Kg/m^2^) | | A (23)  B (20)  C (2) | 22.20  23.40  21.00 | 0.914 | 0.502 |
| Smoking | | A (23)  B (20)  C (2) | 21.20  23.50  41.00 | 1.672 | 0.095 |
| Alcohol | | A (23)  B (20)  C (2) | 21.70  25.25  19.00 | 0.656 | 0.512 |
| Abdominal operation | | A (23)  B (20)  C (2) | 21.43  25.25  18.50 | 0.964 | 0.335 |
| T2DM | | A (23)  B (20)  C (2) | 22.96  23.25  21.00 | -0.045 | 0.964 |
| Cardiovascular disease | | A (23)  B (20)  C (2) | 22.00  24.25  22.00 | 1.310 | 0.190 |
| Hypertension | | A (23)  B (20)  C (2) | 22.39  22.00  40.00 | 0.898 | 0.369 |
| COPD | | A (23)  B (20)  C (2) | 22.96  23.25  21,00 | -0.045 | 0.964 |
| Hepatitis | | A (23)  B (20)  C (2) | 22.50  22.50  33.75 | 1.876 | 0.061 |
| Kidney disease | | A (23)  B (20)  C (2) | 23.48  22.50  22.50 | -0.960 | 0.337 |
| Hyperlipidemia | | A (23)  B (20)  C (2) | 22.50  23.63  22.50 | 0.916 | 0.360 |
| Hemoglobin (g/L) | | A (23)  B (20)  C (2) | 25.67  21.13  11.00 | -1.960 | **0.050** |
| Tumor location | | A (23)  B (20)  C (2) | 24.00  22.63  15.25 | -1.113 | 0.266 |
| NRS2002 | | A (23)  B (20)  C (2) | 23.63  21.63  29.50 | -0.213 | 0.831 |
| LCR | | A (23)  B (20)  C (2) | 24.91  21.00  21.00 | -1.988 | **0.047** |
| ASA score | | A (23)  B (20)  C (2) | 22.57  23.23  25.75 | 0.311 | 0.756 |
| ECOG score | | A (23)  B (20)  C (2) | 20.13  26.60  20.00 | 1.729 | 0.084 |
| Total bilirubin(μmol/L) | | A (23)  B (20)  C (2) | 27.39  16.50  37.50 | -1.871 | 0.061 |
| Direct bilirubin(μmol/L)  A (23)  B (20)  C (2) | | | 26.57  17.38  38.25 | -1.483 | 0.138 |
| ALT(IU/L) | | A (23)  B (20)  C (2) | 21.02  23.13  44.50 | 1.468 | 0.142 |
| AST(IU/L) | | A (23)  B (20)  C (2) | 22.50  21.45  44.25 | 0.704 | 0.481 |
| Prealbumin(g/L) | | A (23)  B (20)  C (2) | 28.76  15.98  27.00 | -2.736 | **0.006** |
| Albumin(g/L) | | A (23)  B (20)  C (2) | 24.17  22.15  18.00 | -0.646 | 0.518 |
| Urea(mmol/L) | | A (23)  B (20)  C (2) | 22.33  23.63  24.50 | 0.367 | 0.713 |
| Creatinine(μmol/L) | | A (23)  B (20)  C (2) | 16.28  29.88  31.50 | 3.433 | **0.001** |
| Uric acid(μmol/L) | | A (23)  B (20)  C (2) | 20.65  24.93  30.75 | 1.314 | 0.189 |
| White blood count(10⁹/L)  A (23)  B (20)  C (2) | | | 21.33  25.38  18.50 | 0.713 | 0.476 |
| Neutrophil count (10⁹/L)  A (23)  B (20)  C (2) | | | 20.96  26.20  14.50 | 0.802 | 0.423 |
| Lymphocyte count(10⁹/L)  A (23)  B (20)  C (2) | | | 24.59  20.93  25.50 | -0.702 | 0.483 |
| Hematocrit(%) | | A (23)  B (20)  C (2) | 23.30  21.55  34.00 | 0.111 | 0.911 |
| Platelet count(10⁹/L) | | A (23)  B (20)  C (2) | 21.09  26.80  7.00 | 0.601 | 0.548 |
| APTT(s) | | A (23)  B (20)  C (2) | 21.98  23.43  30.50 | 0.702 | 0.483 |
| PT(s) | | A (23)  B (20)  C (2) | 22.04  23.85  25.50 | 0.524 | 0.600 |
| INR | | A (23)  B (20)  C (2) | 23.65  22.08  24.75 | -0.324 | 0.746 |

Abbreviations: BMI, body mass index; ASA, American Society of Anesthesiologists; ECOG, Eastern Cooperative Oncology Group; COPD, Chronic Obstructive Pulmonary Disease; NRS2002, Nutritional Risk Screening 2002; LCR, Lymphocyte-C-reactive protein Ratio; ALT, alanine aminotransferase; AST, aspartate aminotransferase; T2DM, type 2 diabetes mellitus; APTT, activated partial thromboplasin time; PT, prothrombin time; INR, international normalized ration.

Supplement Table 2-2. Correlation of AL severity with LCR and other factors

| **Factors** | **A vs B(***p***)** | **A vs C(***p***)** | **B vs C(***p***)** | **A vs BC(***p***)** | **AB vs C(***p***)** |
| --- | --- | --- | --- | --- | --- |
| LCR | **0.049** | 0.520 | 0.746 | **0.043** | 0.651 |
| Hemoglobin (g/L) | 0.068 | 0.071 | 0.217 | 0.106 | 0.122 |
| Prealbumin(g/L) | **0.001** | 0.252 | 0.923 | **0.001** | 0.941 |
| Creatinine(μmol/L) | **0.001** | 0.442 | 0.928 | **0.003** | 0.961 |

Note: A, Grade A anastomotic fistula; B, Grade B anastomotic fistula; C, Grade C anastomotic fistula.
