# Supplementary material for: A preoperative prediction model based on Lymphocyte-C-reactive protein ratio predicts postoperative anastomotic leakage in patients with colorectal carcinoma: a retrospective study
Source: BMC Surg. 2022 Jul 23;22:283. doi: 10.1186/s12893-022-01734-5 (PMC9308913; doi:10.1186/s12893-022-01734-5)
Supplement: Supplementary file 3 — Additional file 3: Table S3. Distribution of predictor variables in overall cohorts for model for prediction of AL. Continuous variables are expressed as mean±standard deviation. Categorical variables are expressed as numbers (percentages) of patients. [file 12893_2022_1734_MOESM3_ESM.docx]

Additional file 3: Table S3 Distribution of predictor variables in overall cohorts for model for prediction of AL. Continuous variables are expressed as mean±standard deviation. Categorical variables are expressed as numbers (percentages) of patients

| Factors | Overall cohort | |
| --- | --- | --- |
|  | Anastomotic Leakage n(%) | Non- leakage n(%) |
| Age (years) | 67.11±10.56 | 63.18±11.63 |
| BMI (Kg/m^2^) | 22.65±3.08 | 22.33±2.62 |
| Sex |  |  |
| Male | 37(68.5) | 457(57.8) |
| Female | 17(31.5) | 333(42.2) |
| Smoking |  |  |
| Yes | 10(18.5) | 136(17.2) |
| No | 44(81.5) | 654(82.8) |
| Alcohol |  |  |
| Yes | 10(18.5) | 91(11.5) |
| No | 44(81.5) | 699(88.5) |
| Abdominal operation |  |  |
| Yes | 12(22.2) | 200(25.3) |
| No | 42(77.8) | 590(74.7) |
| T2DM |  |  |
| Yes | 5(9.3) | 79(10.0) |
| No | 49(90.7) | 711(90.0) |
| Cardiovascular disease |  |  |
| Yes | 5(9.3) | 84(10.6) |
| No | 49(90.7) | 706(89.4) |
| Hypertension |  |  |
| Yes | 15(27.8) | 238(30.1) |
| No | 39(72.2) | 552(69.9) |
| COPD |  |  |
| Yes | 4(7.4) | 47(5.9) |
| No | 50(92.6) | 743(94.1) |
| Hepatitis |  |  |
| Yes | 1(1.9) | 32(4.1) |
| No | 53(98.1) | 758(95.9) |
| Kidney disease |  |  |
| Yes | 2(3.7) | 21(2.7) |
| No | 52(96.3) | 769(97.3) |
| Hyperlipidemia |  |  |
| Yes | 1(1.9) | 18(2.3) |
| No | 53(98.1) | 772(97.7) |
| Transfusion history |  |  |
| Yes | 2(3.7) | 48(6.1) |
| No | 52(96.3) | 742(93.9) |
| Bowel preparation |  |  |
| Yes | 53(98.1) | 786(99.5) |
| No | 1(1.9) | 4(0.5) |
| Tumor location |  |  |
| rectum | 39(72.2) | 421(53.3) |
| descending, sigmoid colon | 4(7.4) | 119(15.1) |
| transverse colon | 2(3.7) | 139(17.6) |
| cecum, ascending colon | 9(16.7) | 111(14.0) |
| NRS2002 |  |  |
| ≧3 | 40(74.1) | 330(41.8) |
| ﹤3 | 14(25.9) | 460(58.2) |
| ASA score |  |  |
| Ⅰ | 20(37.0) | 438(55.4) |
| Ⅱ | 25(46.3) | 260(32.9) |
| Ⅲ | 7(12.9) | 86(10.9) |
| Ⅳ | 2(3.8) | 6(0.8) |
| ECOG score |  |  |
| 0 | 3(5.6) | 190(24.1) |
| 1 | 37(68.4) | 349(44.2) |
| 2 | 12(22.2) | 216(27.3) |
| 3 | 1(1.9) | 32(4.1) |
| 4 | 1(1.9) | 3(0.3) |
| Hemoglobin (g/L) |  |  |
| ≧90 | 28(51.9) | 647(81.9) |
| ﹤90 | 26(48.1) | 143(18.1) |
| LCR |  |  |
| ﹥6000 | 5(9.3) | 269(33.4) |
| ≦6000 | 49(90.7) | 521(66.6) |
| Total bilirubin (μmol/L) | 12.13±6.08 | 11.24±6.73 |
| Direct bilirubin (μmol/L) | 3.20±1.57 | 2.78±1.40 |
| ALT(IU/L) | 17.57±11.30 | 17.11±12.34 |
| AST(IU/L) | 18.04±8.50 | 18.49±8.20 |
| Prealbumin(g/L) | 0.18±0.06 | 0.31±0.21 |
| Albumin(g/L) | 38.11±4.01 | 38.64±4.85 |
| Urea(mmol/L) | 5.23±2.20 | 5.09±2.33 |
| Creatinine(μmol/L) | 78.65±15.16 | 77.22±31.32 |
| Uric acid(μmol/L) | 302.79±91.38 | 294.96±90.19 |
| White blood count (10⁹/L) | 6.47±2.52 | 6.20±2.18 |
| Neutrophil count (10⁹/L) | 4.27±2.41 | 3.94±2.00 |
| Lymphocyte count (10⁹/L) | 1.52±0.60 | 1.66±1.20 |
| Hematocrit(%) | 35.90±5.77 | 35.21±14.16 |
| Platelet count (10⁹/L) | 243.34±94.03 | 231.89±90.63 |
| APTT(s) | 32.95±4.21 | 32.40±4.30 |
| PT(s) | 11.40±1.40 | 11.24±1.23 |
| INR | 1.01±0.15 | 0.99±0.11 |
| C-reactive protein (ng/L) | 6.18±3.76 | 3.97±8.29 |

Abbreviations: BMI, body mass index; ASA, American Society of Anesthesiologists; ECOG, Eastern Cooperative Oncology Group; COPD, Chronic Obstructive Pulmonary Disease; NRS2002, Nutritional Risk Screening 2002; LCR, Lymphocyte-C-reactive protein Ratio; ALT, alanine aminotransferase; AST, aspartate aminotransferase; T2DM, type 2 diabetes mellitus; APTT, activated partial thromboplasin time; PT, prothrombin time; INR, international normalized ration.
