# Supplementary material for: A preoperative prediction model based on Lymphocyte-C-reactive protein ratio predicts postoperative anastomotic leakage in patients with colorectal carcinoma: a retrospective study
Source: BMC Surg. 2022 Jul 23;22:283. doi: 10.1186/s12893-022-01734-5 (PMC9308913; doi:10.1186/s12893-022-01734-5)
Supplement: Supplementary file 4 — Additional file 4: Table S4. Uni- and multivariate analysis of preoperative predictors of AL (overall analysis). [file 12893_2022_1734_MOESM4_ESM.docx]

Additional file 4:Table S4. Uni- and multivariate analysis of preoperative predictors of AL (overall analysis).

|  |  | Univariate analysis | Multivariate analysis |  |
| --- | --- | --- | --- | --- |
|  |  | *p* | OR（95% CI） | *p* |
| Age (years) | (≥60/<60) | **0.004** | 2.60(1.17-6.63) | **0.029** |
| Sex | (female/male) | 0.124 |  |  |
| BMI (Kg/m^2^) |  | 0.574 |  |  |
| Smoking | (Yes/No) | 0.806 |  |  |
| Alcohol |  | 0.125 |  |  |
| Abdominal operation | (Yes/No) | 0.612 |  |  |
| T2DM | (Yes/No) | 0.860 |  |  |
| Cardiovascular disease | (Yes/No) | 0.751 |  |  |
| Hypertension | (Yes/No) | 0.715 |  |  |
| COPD | (Yes/No) | 0.664 |  |  |
| Hepatitis | (Yes/No) | 0.420 |  |  |
| Kidney disease | (Yes/No) | 0.648 |  |  |
| Hyperlipidemia | (Yes/No) | 0.838 |  |  |
| Transfusion history | (Yes/No) | 0.475 |  |  |
| Bowel preparation | (Yes/No) | 0.213 |  |  |
| Hemoglobin (g/L) | (≥90/< 90) | **0.010** | 0.33(0.18-0.61) | **0.019** |
| Tumor location | transverse colon descending, sigmoid colon rectum | **0.001** | 1.48(1.08-2.08) | **<0.001** |
| NRS2002 | (≥3/<3) | **<0.001** | 2.47(1.28-4.98) | **0.009** |
| LCR | (>6000/≤6000) | **<0.001** | 0.22(0.08-0.53) | **0.002** |
| ASA score | (Ⅰ/Ⅱ/Ⅲ/IV) | **0.024** | 1.48(0.94-2.31) | 0.089 |
| ECOG score | (0/1/2/3/4) | **0.002** | 0.76(0.49-1.17) | 0.200 |
| Total bilirubin(μmol/L) |  | 0.343 |  |  |
| Direct bilirubin(μmol/L) | | **0.035** | 1.20(0.99-1.43) | 0.054 |
| ALT(IU/L) |  | 0.773 |  |  |
| AST(IU/L) |  | 0.705 |  |  |
| Prealbumin(g/L) |  | 0.631 |  |  |
| Albumin(g/L) |  | 0.436 |  |  |
| Urea(mmol/L) |  | 0.658 |  |  |
| Creatinine(μmol/L) |  | 0.740 |  |  |
| Uric acid(μmol/L) |  | 0.538 |  |  |
| White blood count(10⁹/L) | | 0.390 |  |  |
| Neutrophil count (10⁹/L) | | 0.247 |  |  |
| Lymphocyte count(10⁹/L) | | 0.404 |  |  |
| Hematocrit(%) |  | 0.937 |  |  |
| Platelet count(10⁹/L) |  | 0.603 |  |  |
| APTT(s) |  | 0.541 |  |  |
| PT(s) |  | 0.283 |  |  |
| INR |  | 0.102 |  |  |
| C-reactive protein(ng/L) | | 0.117 | 1.031(0.990-1.060) | 0.040 |

Abbreviations: BMI, body mass index; ASA, American Society of Anesthesiologists; ECOG, Eastern Cooperative Oncology Group; COPD, Chronic Obstructive Pulmonary Disease; NRS2002, Nutritional Risk Screening 2002; LCR, Lymphocyte-C-reactive protein Ratio; ALT, alanine aminotransferase; AST, aspartate aminotransferase; T2DM, type 2 diabetes mellitus; APTT, activated partial thromboplasin time; PT, prothrombin time; INR, international normalized ration.
