# Supplementary material for: A preoperative prediction model based on Lymphocyte-C-reactive protein ratio predicts postoperative anastomotic leakage in patients with colorectal carcinoma: a retrospective study
Source: BMC Surg. 2022 Jul 23;22:283. doi: 10.1186/s12893-022-01734-5 (PMC9308913; doi:10.1186/s12893-022-01734-5)
Supplement: Supplementary file 5 — Additional file 5: Table S5. Uni- and multivariate analysis of preoperative predictors of AL (right hemi colectomy). [file 12893_2022_1734_MOESM5_ESM.docx]

Additional file 5:Table S5. Uni- and multivariate analysis of preoperative predictors of AL (right hemi colectomy).

|  |  | Univariate analysis | Multivariate analysis |  |
| --- | --- | --- | --- | --- |
|  |  | *p* | OR（95% CI） | *p* |
| Age (years) | (≥60/<60) | 0.548 |  |  |
| Sex | (female/male) | 0.442 |  |  |
| BMI (Kg/m^2^) |  | 0.734 |  |  |
| Smoking | (Yes/No) | 0.359 |  |  |
| Alcohol |  | 0.956 |  |  |
| Abdominal operation | (Yes/No) | 0.612 |  |  |
| T2DM | (Yes/No) | 0.978 |  |  |
| Cardiovascular disease | (Yes/No) | 0.086 |  |  |
| Hypertension | (Yes/No) | 0.326 |  |  |
| COPD | (Yes/No) | 0.474 |  |  |
| Hepatitis | (Yes/No) | 0.374 |  |  |
| Kidney disease | (Yes/No) | 0.382 |  |  |
| Hyperlipidemia | (Yes/No) | 0.617 |  |  |
| Transfusion history | (Yes/No) | 0.698 |  |  |
| Bowel preparation | (Yes/No) | 0.177 |  |  |
| Hemoglobin (g/L) | (≥90/< 90) | 0.410 |  |  |
| NRS2002 | (≥3/<3) | **0.038** | 5.99(0.93-6.98) | **0.011** |
| LCR | (>6000/≤6000) | **0.018** | 0.12(0.01-0.84) | **0.039** |
| ASA score | (Ⅰ/Ⅱ/Ⅲ/IV) | **0.001** | 1.99(0.85-4.75) | **0.012** |
| ECOG score | (0/1/2/3/4) | 0.219 |  |  |
| Total bilirubin(μmol/L) |  | 0.950 |  |  |
| Direct bilirubin(μmol/L) | | 0.187 |  |  |
| ALT(IU/L) |  | 0.324 |  |  |
| AST(IU/L) |  | 0.133 |  |  |
| Prealbumin(g/L) |  | 0.773 |  |  |
| Albumin(g/L) |  | 0.929 |  |  |
| Urea(mmol/L) |  | 0.278 |  |  |
| Creatinine(μmol/L) |  | 0.724 |  |  |
| Uric acid(μmol/L) |  | 0.184 |  |  |
| White blood count(10⁹/L) | | 0.601 |  |  |
| Neutrophil count (10⁹/L) | | 0.653 |  |  |
| Lymphocyte count(10⁹/L) | | 0.769 |  |  |
| Hematocrit(%) |  | 0.744 |  |  |
| Platelet count(10⁹/L) |  | **0.048** | 1.01(1.00-1.01) | 0.073 |
| APTT(s) |  | 0.610 |  |  |
| PT(s) |  | 0.112 |  |  |
| INR |  | **0.013** | 3.49(0.04-5.62) | **0.046** |
| C-reactive protein(ng/L) | | 0.440 |  |  |

Abbreviations: BMI, body mass index; ASA, American Society of Anesthesiologists; ECOG, Eastern Cooperative Oncology Group; COPD, Chronic Obstructive Pulmonary Disease; NRS2002, Nutritional Risk Screening 2002; LCR, Lymphocyte-C-reactive protein Ratio; ALT, alanine aminotransferase; AST, aspartate aminotransferase; T2DM, type 2 diabetes mellitus; APTT, activated partial thromboplasin time; PT, prothrombin time; INR, international normalized ration.
